# Database Analysis of Eplerenone Use in Japanese Hypertensive Patients in Clinical Practice

**DOI:** 10.1155/2019/3726419

**Published:** 2019-03-03

**Authors:** Shoko Takahashi, Yoichi Ii, Yuji Yamamoto, Aya Ikeda, Yoko Fujimoto

**Affiliations:** ^1^Internal Medicine Medical Affairs, Pfizer Japan Inc., 3-22-7 Yoyogi, Shibuya-ku, Tokyo 151-8589, Japan; ^2^Clinical Statistics, Development Japan, Pfizer Japan Inc., 3-22-7 Yoyogi, Shibuya-ku, Tokyo 151-8589, Japan; ^3^MinaCare Co., Ltd., 5F 2-3-11 Nihonbashi-Honcho, Chuo-ku, Tokyo 103-0023, Japan; ^4^Upjohn Medical Affairs, Pfizer Japan Inc., 3-22-7 Yoyogi, Shibuya-ku, Tokyo 151-8589, Japan

## Abstract

Eplerenone, a mineralocorticoid receptor antagonist (MRA), is available in Japan, but details of its use in clinical settings have not been thoroughly investigated. Thus, this study was aimed at examining the characteristics of eplerenone-prescribed hypertensive patients in Japan, describing the combination patterns of antihypertensive medications, and comparing eplerenone's mean doses with respect to concomitant diseases. Data of 160,992 hypertensive patients who used the same drugs for six months or more were collected from an insurance database from January 1, 2009, to December 31, 2013. The number of MRA-receiving patients among the extracted population was 3,274 (2%). Compared to patients on eplerenone or spironolactone, patients on neither drug had fewer comorbidities. Eplerenone was administered in combination with calcium channel blockers and angiotensin II receptor blockers in 23.1% and as monotherapy in 6.6% of cases. The most frequent initial daily dose of eplerenone was 50 mg/day followed by 25 mg/day irrespective of the presence of a comorbidity. MRA use was as low as 2%, but its use was more frequent in patients with comorbidities compared to that of other antihypertensives. Despite studies showing eplerenone's efficacy and safety in high-risk hypertensive patients with albuminuria, the drug is not widely used.

## 1. Introduction

Drug treatment options for arterial hypertension have increased and some of them have organ-protective effects [1–3]. Mineralocorticoid receptor antagonists (MRAs) are one such class of drugs. Two MRAs are available in Japan, namely, the selective MRA eplerenone and the nonselective MRA spironolactone [2]. Eplerenone has been indicated for the treatment of hypertension in Japan since 2007 and has more recently been indicated for heart failure in 2016. However, the data on eplerenone are often combined with those on spironolactone, an older MRA. Thus, currently, the use of eplerenone itself is not fully delineated. Recently, the results of postmarketing surveillance of eplerenone in Japan were published [4]; the surveillance provided information on the safety and efficacy of the drug in real-world settings. Nonetheless, it did not provide detailed information on different dosages as related to patient backgrounds such as the presence of concomitant diseases including heart failure or renal disease. Information about antihypertensive drug use including that of MRAs, especially eplerenone, would serve as a useful reference for Japanese physicians as well as those in other countries to understand how these drugs are currently used and how their use should be improved. Therefore, in this study, using a nation-wide, multi-institutional database for reimbursement claims of medical costs, the detailed use of eplerenone in Japanese hypertensive patients was investigated. The objectives of this study were to (1) investigate the characteristics of hypertensive patients prescribed eplerenone or spironolactone and compare them against those of patients who did not use either drug, (2) describe the combination patterns of antihypertensive medications among patients prescribed eplerenone, spironolactone, or neither drug, and (3) compare mean doses of eplerenone with respect to concomitant diseases (cardiovascular diseases, diabetes mellitus, heart failure, and renal dysfunction).

## 2. Materials and Methods

### 2.1. Database

This was a retrospective, noninterventional cross-sectional study using patient data registered in an electronic database of corporate insurance claims developed by MinaCare Co., Ltd., Tokyo, Japan. The details of the MinaCare database have been described by Shima et al.; the database is generally consistent with two national databases and useful owing to a low selection bias and large sample size with wide age distribution [5]. The database used is a subject-level database that protects the identity of individuals. MinaCare is allowed to use such anonymized data under the data transfer contract with its client health insurers. We complied with the Ethical Guidelines for Epidemiological Research set by the Japanese Government [6]. The MinaCare database is suitable for this study to investigate the real- world use of antihypertensive medications owing to its large size. It includes regularly updated data of health checkups as well as medical and pharmaceutical claims. The population covered by the database includes workers and their family members in a wide range of age groups below the age of 75 years. Employment-based health insurance covers a variety of industries across the nation but workers in primary industries, such as agriculture, fisheries, and forestry, and self-employed individuals are not included. As of April 2017, the MinaCare database included medical and pharmaceutical claims of approximately 4.8 million individuals. For the last 5 years, the average number of subjects is about 2 million, accounting for approximately 7% of all insured individuals under employment-based health insurance in Japan.

### 2.2. Selection Criteria

The study inclusion criteria were as follows: (1) patients with arterial hypertension identified using the ICD-10 code and disease name replacement code (Supplemental Table 1) and prescribed antihypertensive drugs (Supplemental Tables 2 and 3) in at least one claim record month from January 1, 2009, to December 31, 2013; and (2) males and females aged 20 years or more in the first claim record month as that referred to in (1). There are no exclusion criteria for this study. All types of data excluding those pertaining to medical procedures were extracted. The sample size was determined by the number of subjects who met the selection criteria in the database during the study period.

### 2.3. Definitions

Stable drug treatment was defined as six or more months of treatment with the same drug or the same combination of drugs. Since the dose was not considered, treatment was considered stable even if the dose had been changed. The eplerenone dose considered was that taken in the last month of a six-month phase during the stable drug treatment period. If there were more than one month's meeting the “last month” condition, then the dose in the first such month was considered. Eplerenone-naïve patients were defined as patients without a record of eplerenone prescription for six months or more. The dose considered in eplerenone-naïve patients was that prescribed in the first month of the treatment. The coding used in the database is listed in Supplemental Tables. The primary definitions of diseases were determined using ICD-10 codes and secondary definitions were used in sensitivity analyses (Supplemental Table 1). Primary definitions were set to identify all patients considered to have the diseases of interest and secondary definitions were used to refine the search further and identify the desired patients with more certainty. For example, the primary definition of arterial hypertension consisted of 38 ICD-10 codes or disease name replacement codes and the secondary definition consisted of 18. Sensitivity analyses regarding disease category definition were planned.

### 2.4. Data Analysis

The analysis was based on descriptive statistical methods. No formal statistical inference was used. From each subject, data beginning at 1 month from the first recorded date (index date) were considered in the analysis. In the case of compound drugs, each constituent drug was counted if the drug classes are different, and patients who received compound drugs were counted as many times as the number of constituent drugs of different classes in the tablet. Records from the last month of the stable drug treatment period were used in the following three comparisons: (1) comparison of the backgrounds among hypertensive patients prescribed eplerenone, spironolactone, or neither drug, (2) comparison of concomitant medications (antihypertensive drug class and number) among hypertensive patients prescribed eplerenone, spironolactone, or neither drug, and (3) comparison of eplerenone dosage (mean dose) with respect to different comorbidities. Summary measures included number of observations (N), mean, median, and standard deviation for continuous variables and N and percentages (%) for categorical variables. No explicit imputation method was used to address the missing values.

## 3. Results and Discussion

### 3.1. Background Characteristics of Patients Prescribed Eplerenone, Spironolactone, or Neither Drug

The number of patients with stable treatment using antihypertensive drugs was 160,992. Since the number of patients who met the primary definition but not the secondary definition was small, 123 (0.1%), only the primary definitions of hypertension were analyzed. The percentage of female patients was 38.0% and the overall mean age was 59.8 (SD = 9.74) years. The percentages of male and female patients were almost equal in the overall population [5]. However, the percentage of female patients was about 20% less than that of male patients among hypertensive patients. The percentages of female patients and the mean ages of patients receiving eplerenone, spironolactone, and neither drug but other antihypertensive drugs are listed in Table 1. Four patients were prescribed both eplerenone and spironolactone. The number of patients receiving an MRA (eplerenone or spironolactone) was 3,274 (2.0%). Of these, 1204 (0.75%) received eplerenone and 2074 (1.3%) received spironolactone.

The most frequent comorbidity among all patients was diabetes (34.9%) followed by cardiovascular disease (32.8%), heart failure (8.4%), and renal disease (7.3%) (Table 2). Compared to patients on stable treatment with eplerenone or spironolactone, patients who received neither drug had a lower percentage of comorbidities including cardiovascular disease, diabetes, or heart failure. In particular, the prevalence of heart failure (7.8%) was significantly lower in patients who received neither drug than in those who received eplerenone (27.9%) or spironolactone (48.1%) (Table 1). Among the patients on eplerenone, the numbers of patients categorized with primary and secondary definitions of comorbidities were similar in the sensitivity analyses, except in the case of renal disease. For renal disease, the secondary definition consisted of “renal failure” only, which was present in 25 patients (2.1%). In contrast, renal disease was identified in 135 patients (11.2%) when the primary definition was applied. Since the number of patients identified using the secondary definition was quite small, the comparison of the results achieved using primary and secondary definitions was considered unreliable and not performed.

### 3.2. Comparison of Concomitant Medication among Patients Prescribed Eplerenone, Spironolactone, or Neither Drug

Among patients who were on stable antihypertensive treatment, the majority were treated by monotherapy (47.1%) followed by dual therapy (38.9%), triple therapy (11.2%), and quadruple therapy (2.4%). Further, in the same population, the most frequent combination of antihypertensive drugs was dual therapy with a calcium channel blocker (CCB) and an angiotensin II receptor blocker (ARB) (25.8%), followed by CCB monotherapy (22.3%) and ARB monotherapy (19.7%) (Table 3), accounting for 67.8% of all antihypertensive combinations. Triple therapy with a CCB, an ARB, and a diuretic accounted for 4.4% of all antihypertensive combinations. The most frequent combination therapy involving an MRA was triple therapy with a CCB, an ARB, and eplerenone (0.2%) or spironolactone (0.1%).

Among patients on stable treatment with eplerenone, the most common treatment was triple therapy (37.1%), followed by dual therapy (24.3%) and quadruple therapy (21.8%); more extensive regimens were used in 10.1% of patients. The most frequent combination treatments involving eplerenone were triple therapy with a CCB, an ARB, and eplerenone (23.1%), followed by dual therapy with a CCB and eplerenone (13.0%). The percentage of each of the other combinations was less than 7.0%. Similarly, for spironolactone, the most frequent combination treatments were triple therapy (34.4%), followed by dual therapy (26.8%) and quadruple therapy (23.3%); more extensive regimens were used in 6.4% of patients. Among patients on eplerenone, 80 (6.6%) were on monotherapy. As for spironolactone, 189 (9.1%) were on monotherapy. The percentage of patients receiving MRA monotherapy was very small; the baseline characteristics of these patients were examined. The comorbidities in patients on eplerenone monotherapy were as follows: cardiovascular disease (43.8%), diabetes (36.3%), heart failure (21.3%), and renal disease (7.5%). Patients on spironolactone monotherapy showed a similar distribution of comorbidities.

Among eplerenone-naïve patients, 137 (12.3%) were on monotherapy. Among these patients, the most frequent comorbidity was cardiovascular disease (47.9%), followed by diabetes (40.5%) and heart failure (29.3%). In the same population, the most frequent initial daily dose of eplerenone was 50 mg/day followed by 25 mg/day (Table 4) and the mean dose (SD) was 42.7 (17.24) mg/day.

### 3.3. Discussion

This study investigated the usage trends of eplerenone in Japan. It showed that the combined use of MRAs, eplerenone and spironolactone, was approximately 2.0% among Japanese patients with hypertension, by using the MinaCare database. Despite studies having shown the efficacy and safety of eplerenone even in high-risk hypertensive patients with albuminuria, eplerenone is not widely used relative to other antihypertensive drugs [7, 8]. Use of eplerenone is allowed in hypertensive patients with either diabetes or microalbuminuria; however, it is contraindicated for hypertensive patients with type 2 diabetes with microalbuminuria. This may be one reason that the usage of eplerenone is limited despite the organ- and blood vessel-protective effects [1–3]. It is speculated that MRAs are used as a last resort to control blood pressure. The prevalence of treatment-resistant hypertension has been reported to be 6.3-21.8% in Japanese hypertensive patients [9, 10]. The majority of the patients with treatment-resistant hypertension appear not to be treated with an MRA and there seem to be scope for the use of MRAs in those patients.

A comparison of patients on MRA with those on other antihypertensive drugs showed that prevalence of the comorbidities such as cardiovascular disease, diabetes, and heart failure was high in patients on MRA. The difference in the prevalence of heart failure was particularly high, with the prevalence being 27.9%, 48.1%, and 7.8% in patients on eplerenone, spironolactone, or neither drug, respectively (Table 1). This suggested that when an MRA is used, it was likely to be chosen when patients had a comorbidity especially heart failure. Among the MRAs used, higher prevalence of comorbid heart failure was reported in patients using spironolactone compared to eplerenone. The reason for this preference could not be explored in this study, but it might be because spironolactone is indicated for edematous conditions in patients with congestive heart failure whereas eplerenone did not have an indication for heart failure at the time when this study data were recorded in Japan. In addition, spironolactone has been available for more than 50 years in Japan and health care providers are used to prescribing it, although more adverse effects are expected with spironolactone due to the difference in selectivity for mineralocorticoid receptors and androgen or progesterone receptors [11].

Treatment of hypertension was largely based on two classes of drugs, CCBs and ARBs. These two classes were used as baseline drugs for the treatment of hypertension. When a triple therapy was chosen, another class was often added to the therapy. MRAs were used in a similar way. The percentage of patients receiving eplerenone monotherapy among patients on stable treatment with eplerenone was less than 10%. Many of the patients receiving eplerenone monotherapy had comorbidity such as cardiovascular disease, diabetes, and/or heart failure. The comorbidities in patients on spironolactone monotherapy were similar to those in patients on eplerenone. Although treatment-resistant hypertension was not investigated in this study, comorbid cardiovascular disease was significantly associated with treatment-resistant hypertension [12], which was probably treated using an MRA.

Among eplerenone-naïve patients, those receiving eplerenone monotherapy accounted for 12.3%. The postmarketing surveillance (PMS) of eplerenone in Japan was conducted from May 2008 to April 2012; it revealed that among patients on eplerenone, 25.7% were on monotherapy [4]. It could be speculated that the discrepancy between the eplerenone use in the PMS and this study could be due to the differences in the patients' age. The mean age of patients on eplerenone in the PMS (standard deviation) was 67.6 (12.8) years while the modal age range was 55 to 65 years in this study. Since the subjects in the PMS were older, it might be possible that they were more likely to be on monotherapy to avoid hypotension due to multiple drugs. The possible selection bias that could have occurred on both the physician and patient sides should be considered during analysis and interpretation to derive optimal benefit from approaches taken in PMS [13]. The median initial daily dose of eplerenone by comorbidity was 50 mg/day for all comorbid conditions (Table 5). The initial dose of eplerenone for the treatment of hypertension should be 50 mg once daily and 25 mg once daily if it is used in combination with a CYP3A4 inhibitor per the package insert [14]. Eplerenone has been indicated for chronic heart failure in Japan since 2016 and the initial dose indicated is 25 mg once daily for heart failure patients without renal impairment. However, at the time of data collection, eplerenone was only indicated for the treatment of hypertension.

### 3.4. Limitations

Using claims data to investigate issues is subject to several limitations that may affect the validity and reproducibility of results. The MinaCare database includes data of corporate employees and their dependents covered by its employment-based health insurance. Due to the Japanese insurance system, whereby people aged 75 years and more have to change their insurance to elderly care insurance, the database does not include this age group (selection bias). This may limit the generalizability of the study. In this study, we defined diseases based on ICD-10 diagnostic codes. However, coding may not be accurately recorded, the diagnosis may be missed in some cases, and different professionals may have different coding patterns, which may affect the results of the study. Regardless, the database is useful and provides the best available data to investigate the actual conditions of diagnosis and treatment of hypertension. Since the number of patients identified using the secondary definition of renal disease was quite small, comparison of the results achieved using primary and secondary definitions was not performed.

## 4. Conclusions

In this study, the usage of eplerenone among hypertensive patients in Japan was investigated. MRA use was considered as generally low in patients with hypertension but it is likely used more in patients with concomitant diseases such as cardiovascular disease, diabetes, and heart failure. Eplerenone was most frequently used as part of triple therapy with a CCB and an ARB. The information on antihypertensive drugs, including MRA use, especially that of eplerenone, would serve as a useful reference for physicians treating arterial hypertension worldwide.

## Figures and Tables

**Table 1:** Characteristics of the study participants: patients on eplerenone, spironolactone, or other antihypertensive drugs during the last claim month of stable treatment (≥ six months of treatment with the same drugs, N = 160,992*∗*).

Characteristic	Patients on eplerenone	Patients on spironolactone	Patients on other antihypertensive drugs
	(N = 1,204)	(N = 2,074)	(N = 157,718)
Age (years), Mean (SD)	57.3 (10.7)	59.8 (10.9)	59.8 (9.7)
Female, N (%)	331 (27.5)	876 (42.2)	59,952 (38.0)
Comorbidities, N (%)			
Cardiovascular disease	578 (48.0)	1,239 (59.7)	51,046 (32.4)
Diabetes	556 (46.2)	1,111 (53.6)	54,553 (34.6)
Heart failure	336 (27.9)	997 (48.1)	12,256 (7.8)
Renal disease	135 (11.2)	247 (11.9)	11,295 (7.2)
Concomitant antihypertensive drugs, N (%)			
Beta blocker	375 (31.1)	828 (39.9)	21,884 (13.9)
Diuretics	317 (26.3)	896 (43.2)	18,352 (11.6)
Eplerenone	1,204 (100.0)	4 (0.2)	0(0)
Spironolactone	4 (0.3)	2,074 (100.0)	0(0)
ACE inhibitor	103 (8.6)	358 (17.3)	10,596 (6.7)
Alpha blocker	81 (6.7)	86 (4.1)	5,568 (3.5)
CCB	816 (67.8)	854 (41.2)	107,496 (68.2)
ARB	770 (64.0)	947 (45.7)	99,840 (63.3)
Others	25 (2.1)	13 (0.6)	976 (0.6)

ACE inhibitor: angiotensin-converting-enzyme inhibitor, CCB: calcium channel blocker, and ARB: angiotensin II receptor blocker. *∗*Note: four patients were prescribed both eplerenone and spironolactone.

SD: standard deviation.

**Table 2:** Concomitant antihypertensives received for concomitant diseases: patients on all antihypertensive drugs during the last claim month of stable treatment (≥ six months of treatment with the same drugs).

	Total N	Cardiovascular disease	Diabetes	Heart failure	Renal disease
		N (%)	N (%)	N (%)	N (%)
All	160,996*∗*	52,863 (32.8)	56,220 (34.9)	13,589 (8.4)	11,677 (7.3)
Beta blocker	23,087	12,429 (53.8)	9,164 (39.7)	5,648 (24.5)	1,900 (8.2)
Diuretics	19,565	7,380 (37.7)	7,879 (40.3)	2,988 (15.3)	1,938 (9.9)
Eplerenone	1,208	580 (48.0)	557 (46.1)	336 (27.8)	136 (11.3)
Spironolactone	2,078	1,241 (59.7)	1,112 (53.5)	997 (48.0)	248 (11.9)
ACE inhibitor	11,057	4,898 (44.3)	4,824 (43.6)	1,893 (17.1)	1,167 (10.6)
Alpha blocker	5,735	2,246 (39.2)	2,290 (39.9)	580 (10.1)	779 (13.6)
CCB	109,166	34,733 (31.8)	37,364 (34.2)	7,552 (6.9)	7,247 (6.6)
ARB	101,557	33,256 (32.7)	38,010 (37.4)	8,294 (8.2)	8,419 (8.3)
Others	1,014	438 (43.2)	453 (44.7)	166 (16.4)	265 (26.1)

ACE inhibitor: angiotensin-converting-enzyme inhibitor, CCB: calcium channel blocker, and ARB: angiotensin II receptor blocker.

*∗*Note: four patients who were prescribed both eplerenone and spironolactone have been counted twice.

**Table 3:** Combinations of antihypertensive drugs in order of frequency in all patients who received antihypertensive drugs during the last claim month of stable treatment (≥ six months of treatment with the same drugs; combinations with 0.2% or more are shown).

Combinations	N (%)
CCB+ARB	41,462 (25.8)
CCB	35,946 (22.3)
ARB	31,757 (19.7)
D+CCB+ARB	7,048 (4.4)
D+ARB	4,850 (3.0)
BB+CCB+ARB	4,716 (2.9)
BB+CCB	4,663 (2.9)
ACEi+CCB	4,066 (2.5)
BB	4,061 (2.5)
BB+ARB	2,673 (1.7)
ACEi	2,512 (1.6)
AlphaB+CCB+ARB	1,627 (1.0)
BB+D+CCB+ARB	1,444 (0.9)
D+CCB	927 (0.6)
AlphaB+CCB	908 (0.6)
BB+ACEi+CCB	773 (0.5)
BB+ACEi	764 (0.5)
BB+D+ARB	686 (0.4)
D	680 (0.4)
D+AlphaB+CCB+ARB	517 (0.3)
ACEi+CCB+ARB	508 (0.3)
AlphaB+ARB	419 (0.3)
BB+AlphaB+CCB+ARB	370 (0.2)
AlphaB	345 (0.2)
D+ACEi+CCB	315 (0.2)
EPL+CCB+ARB	278 (0.2)

CCB: calcium channel blocker, ARB: angiotensin II receptor blocker, D: diuretics, BB: beta blocker, ACEi: angiotensin-converting-enzyme inhibitor, AlphaB: alpha blocker, and EPL: eplerenone.

**Table 4:** Initial daily dose of eplerenone in eplerenone-naïve patients.

Dose (mg/day)	N (%)
<25	8 (0.7)
25	396 (35.4)
50	676 (60.5)
100	37 (3.3)
>100	1 (0.1)
All	1118 (100.0)

**Table 5:** Initial daily dose of eplerenone by comorbidities in eplerenone-naïve patients.

	N (%)	Dose (mg/day)			
		Mean (SD)	25th percentile	Median	75th percentile
Cardiovascular disease	535 (47.9)	41.3 (16.55)	25	50	50
Diabetes	453 (40.5)	43.2 (19.80)	25	50	50
Heart failure	328 (29.3)	40.6 (17.04)	25	50	50
Renal disease	113 (10.1)	40.9 (18.84)	25	50	50
All	1118	42.7 (17.24)	25	50	50

SD: standard deviation.

## Data Availability

The database data used to support the findings of this study are included within the article and the supplementary information file.

## Supplementary Materials

Supplementary Materials Supplementary Table 1: disease code. Supplemental Table 2: drug code by drug class.
