# Supplementary material for: Database Analysis of Eplerenone Use in Japanese Hypertensive Patients in Clinical Practice
Source: Int J Hypertens. 2019 Mar 3;2019:3726419. doi: 10.1155/2019/3726419 (PMC6420997; doi:10.1155/2019/3726419)
Supplement: Supplementary Materials — Supplementary Table 1: disease code. Supplemental Table 2: drug code by drug class. [file 3726419.f1.docx]

**Supplementary Material**

Supplemental Table 1 Disease code

|  | **Primary definition: ICD-10 Code** | **Secondary definition used for the sensitivity analysis: ICD-10 code** |
| --- | --- | --- |
| **Heart failure** | I11.0, I50.0, I50.1, I50.9, I13.0, I13.2, CJ4E*, HCSK*, HD64* | I50.0, I50.1, I50.9 |
| **Diabetes** | E10.0, E10.1, E10.2, E10.3, E10.4, E10.5, E10.6, E10.7, E10.9, E11, E11.0, E11.1, E11.2, E11.3, E11.4, E11.5, E11.6, E11.7, E11.9, E12, E12.0, E12.1, E12.2, E12.3, E12.4, E12.5, E12.6, E12.7, E12.9, E13, E13.0, E13.1, E13.2, E13.3, E13.4, E13.5, E13.6, E13.7, E13.9, E14, E14.0, E14.1, E14.2, E14.3, E14.4, E14.5, E14.6, E14.7, E14.9, O240, O241, O244, O249 | E10.0, E10.1, E10.2, E10.3, E10.4, E10.5, E10.6, E10.7, E10.9, E11, E11.0, E11.1, E11.2, E11.3, E11.4, E11.5, E11.6, E11.7, E11.9, E12, E12.0, E12.1, E12.2, E12.3, E12.4, E12.5, E12.6, E12.7, E12.9, E13, E13.0, E13.1, E13.2, E13.3, E13.4, E13.5, E13.6, E13.7, E13.9, E14, E14.0, E14.1, E14.2, E14.3, E14.4, E14.5, E14.6, E14.7, E14.9 |
| **Hypertension** | I10, I11, I11.0, I11.9, I12, I12.0, I12.9, I13, I13.0, I13.1, I13.2, I13.9, I15, I15.0, I15.1, I15.2, I15.8, I15.9, I67.4, O10, O10.0, O10.1, O10.2, O10.3, O10.4, O10.9, O11, O13, O14, O14.0, O14.1, O14.9, O16, G791, VQED*,　UPPH*, PNLT*, KQP2* | I10, I11, I11.0, I11.9, I12, I12.0, I12.9, I13, I13.0, I13.1, I13.2, I13.9, I15, I15.0, I15.1, I15.2, I15.8, I15.9 |
| **Renal diseases** | N00, N00.0, N00.1, N00.2, N00.3, N00.4, N00.5, N00.6, N00.7, N00.8, N00.9, N01, N01.0, N01.1, N01.2, N01.3, N01.4, N01.5, N01.6, N01.7, N01.8, N01.9, N02, N02.0, N02.1, N02.2, N02.3, N02.4, N02.5, N02.6, N02.7, N02.8, N02.9, N03, N03.0, N03.1, N03.2, N03.3, N03.4, N03.5, N03.6, N03.7, N03.8, N03.9, N04, N04.0, N04.1, N04.2, N04.3, N04.4, N04.5, N04.6, N04.7, N04.8, N04.9, N05, N05.0, N05.1, N05.2, N05.3, N05.4, N05.5, N05.6, N05.7, N05.8, N05.9, N06, N06.0, N06.1, N06.2, N06.3, N06.4, N06.5, N06.6, N06.7, N06.8, N06.9, N07, N07.0, N07.1, N07.2, N07.3, N07.4, N07.5, N07.6, N07.7, N07.8, N07.9, N08, N08.0, N08.1, N08.2, N08.3, N08.4, N08.5, N08.8, N10, N11, N11.0, N11.1, N11.8, N11.9, N12, N13, N13.0, N13.1, N13.2, N13.3, N13.4, N13.5, N13.6, N13.7, N13.8, N13.9, N14, N14.0, N14.1, N14.2, N14.3, N14.4, N15, N15.0, N15.1, N15.8, N15.9, N16, N16.0, N16.1, N16.2, N16.3, N16.4, N16.5, N16.8, N17, N17.0, N17.1, N17.2, N17.8, N17.9, N18, N18.0, N18.8, N18.9, N19, N25, N25.0, N25.1, N25.8, N25.9, N26, N27, N27.0, N27.1, N27.9, N28, N28.0, N28.1, N28.8, N28.9, N29, N29.0, N29.1, N29.8, E27.2, I12.0, I12.9, I12, N99.0, O90.4 | N18, N18.0, N18.8, N18.9 |
| **Other cardiovascular diseases** | I20, I20.0, I20.1, I20.8, I20.9, I21, I21.0, I21.1, I21.2, I21.3, I21.4, I21.9, I22, I22.0, I22.1, I22.8, I22.9, I23, I23.0, I23.1, I23.2, I23.3, I23.4, I23.5, I23.6, I23.8, I24, I24.0,I24.1, I24.8, I24.9, I25, I25.0, I25.1, I25.2, I25.3, I25.4, I25.5, I25.6, I25.8, I25.9, I26, I26.0, I26.9, I27, I27.0, I27.1, I27.2, I27.8, I27.9, I28, I28.0, I28.1, I28.8, I28.9, I30, I30.0, I30.1, I30.8, I30.9, I31, I31.0, I31.1, I31.2, I31.3, I31.8, I31.9, I32, I32.0, I32.1, I32.8, I33, I33.0, I33.9, I34, I34.0, I34.1, I34.2, I34.8, I34.9, I35, I35.0, I35.1, I35.2, I35.8, I35.9, I36, I36.0, I36.1, I36.2, I36.8, I36.9, I37, I37.0, I37.1, I37.2, I37.8, I37.9, I38, I39, I39.0, I39.1, I39.2, I39.3, I39.4, I39.8, I40, I40.0, I40.1, I40.8, I40.9, I41, I41.0, I41.1, I41.2, I41.8, I42, I42.0, I42.1, I42.2, I42.3, I42.4, I42.5, I42.6, I42.7, I42.8, I42.9, I43, I43.0, I43.1, I43.2, I43.8, I44, I44.0, I44.1, I44.2, I44.3, I44.4, I44.5, I44.6, I44.7, I45, I45.0, I45.1, I45.2, I45.3, I45.4, I45.5, I45.6, I45.8, I45.9, I46, I46.0, I46.1, I46.9, I47, I47.0, I47.1, I47.2, I47.9, I48, I49, I49.0, I49.1, I49.2, I49.3, I49.4, I49.5, I49.8, I49.9, I51, I51.0, I51.1, I51.2, I51.3, I51.4, I51.5, I51.6, I51.7, I51.8, I51.9, I52, I52.0, I52.1, I52.8, I60, I60.0, I60.1, I60.2, I60.3, I60.4, I60.5, I60.6, I60.7, I60.8, I60.9, I61, I61.0, I61.1, I61.2, I61.3, I61.4, I61.5, I61.6, I61.8, I61.9, I62, I62.0, I62.1, I62.9, I63, I63.0, I63.1, I63.2, I63.3, I63.4, I63.5, I63.6, I63.8, I63.9, I64, I65, I65.0, I65.1, I65.2, I65.3, I65.8, I65.9, I66, I66.0, I66.1, I66.2, I66.3, I66.4, I66.8, I66.9, I67, I67.0, I67.1, I67.2, I67.3, I67.4, I67.5, I67.6, I67.7, I67.8, I67.9, I68, I68.0, I68.1, I68.2, I68.8, I69, I69.0, I69.1, I69.2, I69.3, I69.4, I69.8, I70, I70.0, I70.1, I70.2, I70.8, I70.9, I71, I71.0, I71.1, I71.2, I71.3, I71.4, I71.5, I71.6, I71.8, I71.9, I72, I72.0, I72.1, I72.2, I72.3, I72.4, I72.8, I72.9, I73, I73.0, I73.1, I73.8, I73.9, I74, I74.0, I74.1, I74.2, I74.3, I74.4, I74.5, I74.8, I74.9, I77, I77.0, I77.1, I77.2, I77.3, I77.4, I77.5, I77.6, I77.8, I77.9, I78, I78.0, I78.1, I78.8 ,I78.9, I79, I79.0, I79.1, I79.2, I79.8 | (None) |

*Disease name replacement code

Supplemental Table 2. Drug code by drug class

| Drug class | Generic Drug Name |
| --- | --- |
| Beta blockers | Acebutolol hydrochloride |
|  | Carteolol hydrochloride |
|  | Propranolol hydrochloride |
|  | Pindolol |
|  | Atenolol |
|  | Arotinolol hydrochloride |
|  | Nadolol |
|  | Bisoprolol fumarate |
|  | Labetalol hydrochloride |
|  | Metoprolol tartrate |
|  | Pindolol |
|  | Propranolol hydrochloride |
|  | Amosulalol hydrochloride |
|  | Nipradilol |
|  | Carteolol hydrochloride |
|  | Tilisolol hydrochloride |
|  | Celiprolol hydrochloride |
|  | Betaxolol hydrochloride |
|  | Carvedilol |
|  | Bevantolol hydrochloride |
| Diuretics | Trichlormethiazide |
|  | Hydrochlorothiazide |
|  | Benzylhydrochlorothiazide |
|  | Triamterene |
|  | Mefruside |
|  | Furosemide |
|  | Meticrane |
|  | Tripamide |
|  | Indapamide |
| Diuretics + Others | Benzylhydrochlorothiazide/Reserpine |
| Diuretics + Angiotensin II receptor blockers | [Losartan](https://eow.alc.co.jp/search?q=losartan&ref=awlj) [potassium](https://eow.alc.co.jp/search?q=potassium&ref=awlj)/Hydrochlorothiazide |
|  | [Candesartan](https://eow.alc.co.jp/search?q=candesartan&ref=awlj) [cilexetil](https://eow.alc.co.jp/search?q=cilexetil&ref=awlj)/Hydrochlorothiazide |
|  | Valsartan/Hydrochlorothiazide |
|  | Telmisartan/Hydrochlorothiazide |
|  | Irbesartan/Trichlormethiazide |
| Mineralocorticoid receptor antagonists | Spironolactone |
|  | Eplerenone |
| Others | Budralazine |
|  | Hydralazine Hydrochloride |
|  | Reserpine |
|  | Methyldopa hydrate |
|  | Aliskiren fumarate |
| Angiotensin-converting-enzyme (ACE) inhibitor | Captopril |
|  | Enalapril maleate |
|  | Alacepril |
|  | Delapril hydrochloride |
|  | Cilazapril hydrate |
|  | Lisinopril hydrate |
|  | Benazepril hydrochloride |
|  | Imidapril hydrochloride |
|  | Temocapril hydrochloride |
|  | Quinapril hydrochloride |
|  | Trandolapril |
|  | Perindopril erbumine |
| Alpha blockers | Clonidine hydrochloride |
|  | Prazosin hydrochloride |
|  | Bunazosin hydrochloride |
|  | Guanabenz acetate |
|  | Urapidil |
|  | Terazosin hydrochloride hydrate |
|  | Doxazosin mesilate |
| Calcium channel blockers | Nicardipine hydrochloride |
|  | Nilvadipine |
|  | Manidipine hydrochloride |
|  | Barnidipine hdrochloride |
|  | Efonidipine hydrochloride ethanolate |
|  | Felodipine |
|  | Cilnidipine |
|  | Aranidipine |
|  | Azelnidipine |
|  | Diltiazem hydrochloride |
|  | Nifedipine |
|  | Nisoldipine |
|  | Nitrendipine |
|  | Benidipine hydrochloride |
|  | Amlodipine besilate |
|  | Amlodipine besilate/Atorvastatin calcium hydrate* |
| Calcium channel blockers + Angiotensin II receptor blockers | Valsartan/Amlodipine besylate* |
|  | Olmesartan medoxomil/Azelnidipine* |
|  | Candesartan cilexetil/Amlodipine besylate* |
|  | Telmisartan/Amlodipine besylate* |
|  | Irbesartan/Amlodipine besylate* |
| Angiotensin II receptor blockers | Losartan potassium |
|  | Candesartan cilexetil |
|  | Valsartan |
|  | Telmisartan |
|  | Olmesartan medoxomil |
|  | Irbesartan |
|  | Azilsartan |

* Indicates compounding tablet
